# Evaluation of Storage Conditions and the Effect on DNA from Forensic Evidence Objects Retrieved from Lake Water

**DOI:** 10.3390/genes15030279

**Published:** 2024-02-23

**Authors:** Muhammad Shahzad, Hanne De Maeyer, Ghassan Ali Salih, Martina Nilsson, Anastasia Haratourian, Muhammad Shafique, Ahmad Ali Shahid, Marie Allen

**Affiliations:** 1 Forensic DNA Typing Laboratory, Centre of Excellence in Molecular Biology, University of the Punjab, Lahore 53700, Pakistan; shahzad.camb@pu.edu.pk (M.S.);; 2 Department of Immunology, Genetics and Pathology, Uppsala University, 751 08 Uppsala, Swedenghassan.salih@igp.uu.se (G.A.S.); martina.nilsson@polisen.se (M.N.);; 3 Centre for Applied Molecular Biology, University of the Punjab, Lahore 53700, Pakistan

**Keywords:** trace evidence, submersion in water, persistence of trace evidence, evidence storage conditions, forensic DNA analysis, STR genotyping, mtDNA sequencing

## Abstract

DNA analysis of traces from commonly found objects like knives, smartphones, tapes and garbage bags related to crime in aquatic environments is challenging for forensic DNA laboratories. The amount of recovered DNA may be affected by the water environment, time in the water, method for recovery, transport and storage routines of the objects before the objects arrive in the laboratory. The present study evaluated the effect of four storage conditions on the DNA retrieved from bloodstains, touch DNA, fingerprints and hairs, initially deposited on knives, smartphones, packing tapes, duct tapes and garbage bags, and submerged in lake water for three time periods. After retrieval, the objects were stored either through air-drying at room temperature, freezing at −30 °C, in nitrogen gas or in lake water. The results showed that the submersion time strongly influenced the amount and degradation of DNA, especially after the longest submersion time (21 days). A significant variation was observed in success for STR profiling, while mtDNA profiling was less affected by the submersion time interval and storage conditions. This study illustrates that retrieval from water as soon as possible and immediate storage through air-drying or freezing before DNA analysis is beneficial for the outcome of DNA profiling in crime scene investigations.

## 1. Introduction

Objects related to a crime, such as smartphones, knives, guns or items wrapped in various types of tape, can potentially contain touch DNA or biological fluids such as blood or saliva [1,2,3]. The items can provide important evidence and may be disposed of in aquatic environments such as lakes, rivers or seas in an attempt to hide their location and make recovery difficult [4]. Forensic divers are, therefore, called in to find suspicious objects and to retrieve them for further investigation. However, exposure to water has been shown to impact DNA profiling negatively [4,5,6]. Degradation and damage to the DNA strands can be accelerated in water due to, for instance, oxidative and microbial exposure [7,8]. Furthermore, the temperature, currents, pH, water flow rates and salinity, as well as the substrate (type of object and surface) and the type of biological fluids, may affect the DNA stability. Several studies have investigated the success of short tandem repeat (STR) analysis from DNA samples recovered from items that have been exposed to water under varying conditions. In general, the studies have revealed that the exposure time in the water, the initial deposited amount of cellular material and the type of biological material directly impact the quality of the recovered DNA [4,5,9,10]. Shed and plucked hairs, for example, are known to contain small amounts of DNA, and it has been observed that >90% of the DNA in the root portion is degraded after 72 h in water [4,11]. In addition, the DNA quantity and quality may also be affected by the post-collection storage and transportation methods. In previous studies, the storage temperature affected the amounts of DNA that can be recovered from different surfaces. For example, higher DNA yields were obtained after storage at −20 °C, and increased storage temperature (room temperature, 37 °C and 50 °C) resulted in reduced DNA quantities [12]. Furthermore, Howlett et al. [13] evaluated storage at the same four different temperatures and observed that DNA storage at −20 °C slows DNA degradation and best preserves the DNA.

The current practice in Sweden is to keep the object in a box with the same water from the recovery area during transportation to the laboratory since it is considered the optimal way of handling evidence for some types of forensic analysis (e.g., digital forensics). However, due to the current low success rate of DNA analysis, other more effective storage methods may be available that preserve the DNA better. In the present study, the effects of different storage conditions on DNA quantity and quality in relevant forensic samples placed on evidence objects recovered from water were evaluated. The disposal and recovery of objects from the water were performed in collaboration with forensic divers at The Marine Unit of Police in Sweden. Knives, smartphones, garbage bags and pieces of tape were utilised in the experiments. The simulated evidence objects were prepared with touch DNA (palm and fingerprints), blood and hairs and the materials were submerged in lake water for 2, 7 and 21 days. Here, four different storage conditions, including air-drying at room temperature, freezing at −30 °C, drying in nitrogen gas and submersion in lake water from the site of recovery, were evaluated. The effects of water exposure and the storage conditions after retrieval were evaluated using real-time DNA quantification, STR analysis and sequencing of mitochondrial DNA (mtDNA) of the different samples.

## 2. Materials and Methods

### 2.1. Samples

The DNA samples used in the present study were obtained from epithelial cells (touch DNA and fingerprints), blood and plucked hairs from three male individuals. Informed consent was obtained from the individuals who provided samples (one individual for each type of sample) and reference saliva samples to evaluate the downstream analysis further. The object types (knives, smartphones, garbage bags, packing tapes, duct tapes) and sample types (blood stains, epithelial cells, fingerprints and hairs) were chosen to be of forensic importance and mimic typical forensic samples that are commonly found and may be retrieved during diving after a crime. The surface of all objects was first decontaminated by submersion into the surface disinfectant DAX Ytdesinfektion Plus^®^ (Opus Health Care AB, Malmö, Sweden) for 20 min, followed by rinsing with water and air-drying before the deposition of the biological materials.

Supplementary S1, Table S1 shows that 56 items for each object type (knives, smartphones, garbage bags and pieces of tape) were prepared in a controlled environment. The volunteers providing touch DNA and fingerprints were asked not to wash their hands 1 h before the deposits. Touch DNA was placed on the handle of each knife (shaft) by holding it in the hand for 10 s with a similar pressure for all objects. Furthermore, each knife blade was stained with 50 µL of blood and left to dry at room temperature. Fingerprints were placed on the front screen and the back side of a smartphone by pressing the index finger for 5 s with similar pressure for all objects. A 10 × 20 cm^2^ area of a black garbage bag was prepared with different samples. One fingerprint was deposited on the garbage bag and left uncovered. An additional fingerprint was placed on the adhesive side of a piece of packing tape (5 × 5 cm^2^) and attached to the garbage bag. In addition, a piece of duct tape (5 × 5 cm^2^) was attached with one single plucked hair adhered to the glue with the shaft kept outside the tape. The deposition of the touch DNA and fingerprints was conducted 2 days before water submersion and left at room temperature. The sample positions were marked with a waterproof pen on all objects to facilitate subsequent sampling. Four biological replicates were prepared on the objects, which were divided into groups for five different exposure times in water: no submersion, 30 min submersion and objects submerged in water for 7, 14 or 21 days (Supplementary S1, Table S1).

### 2.2. Submersion and Storage

The prepared objects were submersed in Lake Mälaren in Stockholm at a depth of 4 m, approximately 60 m from the shore. The mean water temperature at the time of submersion was 3 °C. The objects were placed underwater for recovery after specific time intervals of 30 min and 2, 7 and 21 days. The objects were tied to the bottom of plastic net baskets using zip cable ties to prevent movement of the objects and facilitate easy retrieval by the forensic divers. Upon recovery, the objects were placed in four different storage conditions immediately at the dive site to investigate the four storage methods before sampling: air-drying at room temperature, freezing at −30 °C, placement in a container with nitrogen gas, and submersion in a plastic box with water collected from the dive site. The objects were kept in the storage environment for 2 days. The no-submersion and 30 min groups were air-dried at room temperature.

### 2.3. DNA Sampling, Extraction and Quantification

The objects were removed from the storage conditions and dried at room temperature for 2 h if wet. The deposited traces were collected with a single cotton tip swab (Selefa, OneMed, Helsinki, Finland) moistened with 0.9% NaCl. The swabs were dried and placed in BioPack bags (Nordkrim AB, Helsingborg, Sweden) until extraction. A few of the garbage bag pieces holding the adhesive packing tape and duct tape were placed in a freezer at −30 °C for an additional 2 days to facilitate the release of the adhesive tape. Single hairs were removed from the duct tape using forceps.

As the Swedish National Forensic Laboratory use Chelex^®^ extraction, this method was chosen for all samples collected with swabs to fully simulate the results obtained in casework analysis. The whole swab heads were used for DNA extraction using Chelex^®^ 100 resin (Bio-Rad Laboratories, Hercules, CA, USA) with some modifications for the different types of traces [14]. The swabs with blood traces were incubated using 20% Chelex^®^ 100 resin, and the final volume of the extract was 1 mL. The touch DNA, fingerprint traces and the buccal swabs from the reference samples (positive controls) were extracted using 5% Chelex^®^ 100 in a final extract volume of 170 µL. The hair samples were first washed in 1% SDS solution (Invitrogen, Waltham, MA, USA), and the entire hairs (shaft and root part) were digested using a simple and efficient extraction protocol for hairs with or without roots [11]. Hairs were placed in a lysis buffer of 212 µL containing 1X PCR buffer (Applied Biosystem, Waltham, MA, USA), 240 µg/mL proteinase K (Qiagen, Hilden, Germany) and 33 mM dithiothreitol (DTT; Qiagen) at 56 °C for 7.5 h, followed by 96 °C for 10 min. No further purification was performed of extracted DNA samples (n = 392) prior to storage at −20 °C until further analysis.

The extracted DNA samples, the reagent blanks (negative controls for each extraction batch) and a PCR negative control, were quantified using the Quantifiler^TM^ Trio DNA Quantification kit (Applied Biosystems, Waltham, MA, USA) according to the manufacturer’s instructions. The standard curve had a linear range of 0.005 ng/µL–50 ng/µL, and the run was performed using the QuantStudio^®^5 Real-Time PCR system (Applied Biosystems). Each sample was run in triplicate, and the mean of the technical replicates was calculated for the DNA concentration for each sample category. The Quantifiler™ Trio DNA Quantification Kit amplifies three DNA targets, one male-specific and two autosomal targets, with different amplicon lengths (80 versus 214 bp) used to calculate a degradation index (DI). The mean DI values among the replicates were also calculated. Finally, the 1.5 × interquartile rule determined outliers for all sample categories before calculating the average DNA concentration and total amount of DNA recovered in ng. All quantification data for the different sample categories with four biological and three technical replicates (including standard deviations) are shown in Supplementary S2, Tables S6–S12. Furthermore, the average DNA concentration for samples was grouped and evaluated based on the type of biological sample, type of object, storage condition and submersion time (Supplementary S1, Table S2).

### 2.4. Amplification, Capillary Electrophoresis and STR Analysis

One sample from each sample category was selected for further STR analysis, and the biological replicate (out of four) with the DNA concentration closest to the median value was used, as shown in Supplementary S2, Tables S6–S12. Using this criterium, 98 samples were amplified using the GlobalFiler™ IQC PCR Amplification Kit (Applied Biosystems) with the Veriti^®^ Thermal Cycler (Applied Biosystems). A 32-cycle protocol was followed for optimal amplification from low template DNA [15]. The total extraction volume differed for the various biological materials and was adjusted during the PCR set-up with different DNA input volumes for easier comparison of DNA concentrations. The amplified PCR products (1 μL) were size-separated on a SeqStudio™ Genetic Analyzer (Applied Biosystems) following standard settings and the GeneScan™ 600 LIZ™ Size Standard (Applied Biosystems) with a resolution range of 60–460 bp. A negative control, containing the reagents only, was included in each STR amplification run and the control was analysed together with the samples from each PCR setup. The STR genotypes were determined using the GeneMapper™ software version 6 (Applied Biosystems), and the analytical threshold was set to 50 RFUs. The obtained profiles were compared with the DNA profiles of the sample donors to evaluate the presence of allele drop-ins or drop-outs and potential contamination. Based on the obtained STR profiles, the correctly recovered alleles (in agreement with the respective reference profile from the donor) were calculated and summarised as the percentage of interpretable alleles of the STR profile as a whole. Then, the obtained STR profiles were categorised into three groups: >75% complete STR profile, 50–75% recovered STR profile and <50% markers in the STR profile (Supplementary S2).

### 2.5. mtDNA Sequencing

All hair samples (n = 56) were analysed using mtDNA Sanger sequencing. In addition, samples that generated an STR profile with <75% of the alleles were analysed for mtDNA (n = 70), excluding the hairs. In addition, the biological replicates with the lowest recovered total DNA in all storage conditions for 2, 7 and 21 days of submersion (n = 72) were also mtDNA sequenced. In total, 198 samples were sequenced for one mtDNA region (Supplementary S2). Amplification of the hypervariable region I (HVI, np 16128–16348) was performed using forward and reverse primers (F 5′-ggtaccataaatacttgaccacct-3′ and R 5′-gactgtaatgtgctatgtacggtaaa-3′) in a total volume of 50 μL [16]. The PCR reaction contained 25 μL of AmpliTaq Gold^TM^ 360 master mix (Applied Biosystems) with an additional 1.6 U of Taq Gold polymerase (Applied Biosystems), 0.2 μM of each primer, 0.15 mg/mL bovine serum albumin (BSA, Thermo Fisher Scientific, Waltham, MA, USA) and 15 μL of DNA extract and nuclease-free water. The thermal cycler conditions were incubation for 10 min at 95 °C, followed by 40 cycles of 95 °C for 30 s, 60 °C for 30 s and 72 °C for 30 s. The final extension step was performed at 72 °C for 10 min. The success of the PCR reaction was determined using gel electrophoresis of each PCR product using 2% (*w*/*v*) agarose (BioNordika, Solna, Sweden) with SYBR™ Safe DNA Gel Stain (Invitrogen). All pre-PCR steps were performed in a clean room, and the PCR set-up included amplification of reagent blanks to assess potential contamination.

Purification of PCR products was performed with 5 μL of amplified product using 2 μL of enzyme mixture containing 1 unit of exonuclease I and 2 units of shrimp alkaline phosphatase enzymes (Thermo Fisher Scientific). The reactions were incubated at 37 °C for 15 min and 80 °C for 15 min. Sanger sequencing of HVI was performed in a forward and reverse direction using the BigDye^®^ Terminator v.1.1 Cycle Sequencing Kit (Applied Biosystem). The final reaction volume was 10 μL, which contained 1.6 pmol of the sequencing primer (same as PCR primers), 1.5 μL of purified PCR product, 1 μL of BigDye mix, 2 μL of 5X sequencing buffer and 5 μL nuclease-free water. The cycling PCR reaction was performed on a Veriti^®^ Thermal Cycler (Applied Biosystems) with a first incubation at 95 °C for 30 s followed by 35 cycles at 94 °C for 25 s, 50 °C for 15 s and 60 °C for 2 min. Residual fluorescent nucleotides, salts and other low-molecular-weight materials were removed using an EdgeBio semi-skirt 96-well column plate according to the manufacturer’s instructions (Edge Biosystems, Inc., San Jose, CA, USA) before samples were subjected to capillary electrophoresis on a SeqStudio™ Genetic Analyzer (Applied Biosystems). The mtDNA sequences were aligned to the revised Cambridge Reference Sequence (rCRS) using the Sequencher v. 5.0 Build 7082 software (Gene Codes Corporation, Ann Arbor, MI, USA) to evaluate if an interpretable mtDNA sequence was obtained and whether the sequence was concordant with the mtDNA profile of the sample donor. For all pre-PCR procedures (sampling, extraction, quantification and amplification), personal protective clothing such as a disposable coat, hair net, face mask, double gloves and footwear covers were used.

### 2.6. Statistical Analysis

Microsoft^®^ Excel (version 16.66.1) was used to analyse the obtained quantification data as well as to calculate the average total DNA and outliers for all samples (Supplementary S2, Tables S6–S12). The STR analysis data were compiled in Excel, including calculations of the total allele recovery in % and the average peak heights for the different samples (Supplementary S1, Table S3). RStudio^®^ (version 1.3.1093) was used to produce all figures, heatmaps and the *p*-value table with the Wilcoxon test (Supplementary S1, Table S5) using the following packages: stats, tidyverse, dplyr, ggplot2, scales, ggh4x and readr.

## 3. Results

Different combinations of biological materials and objects were evaluated using blood, touch DNA and hairs on knives, smartphones, garbage bags, packing tapes and duct tapes. Because each of the knives and the smartphones were prepared with two types of simulated evidence, a total of 280 items (56 objects of seven types) and 392 DNA samples (56 objects, seven simulated evidence types) were evaluated (Supplementary S1, Table S1). Touch DNA in the form of fingerprints was placed on the front and back surfaces of a smartphone and on a piece of a garbage bag. The garbage bag was also prepared with fingerprints under a piece of packing tape, and plucked hairs were mounted under duct tape. One set of objects was submersed in lake water and kept there for three different time intervals (2, 7 and 21 days) to evaluate the persistence of DNA. An additional set of objects was submerged for 30 min or not submersed to evaluate the initial DNA contents in the simulated evidence. Moreover, four storage conditions were evaluated for their effect on DNA persistence. The storage methods were air-drying at room temperature, freezing at −30 °C, drying in a container with nitrogen gas and submersion in lake water from the dive site. The effects of object type, evidence type, exposure time in lake water and object storage method were evaluated using DNA quantification, STR analysis and mtDNA sequencing.

### 3.1. DNA Quantification and Degradation

**Figure 1:**
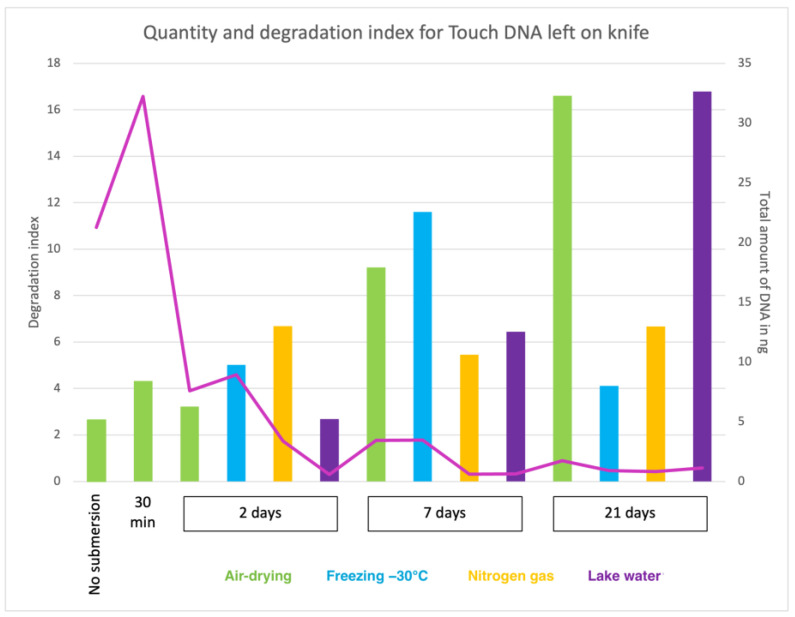
The pink curve displays the total amount of recovered DNA (in ng) from touch DNA deposited on knife shafts with the corresponding value on the Y-axis (right). The degradation index (DI) is shown on the Y-axis (left), and the corresponding values are represented by the columns coloured representing four storage methods: air drying (green), freezing −30 °C (blue), Nitrogen gas (yellow) and lake water (purple). The X-axis represents the submersion times included in the study: no submersion (dry reference), 30 min, 2 days, 7 days and 21 days.

All real-time PCR quantification values are compiled in Supplementary S2 and displayed in different tables based on the type of samples deposited on various objects (seven categories). The tables contain DNA quantification results for all biological and technical replicates, outliers and the average DNA quantities recovered (without outliers) for all samples. In general, a decrease in DNA concentration was observed with longer submersion time. Furthermore, when analysing the DI, an increase in degradation was generally observed with longer submersion time. Thus, the overall results correlated well with the submersion time and an inverse correlation between low DNA amounts and high degradation with more extended time. Still, a considerable variation in DNA concentration and degradation was observed (Figure 1).

**Figure 2:**
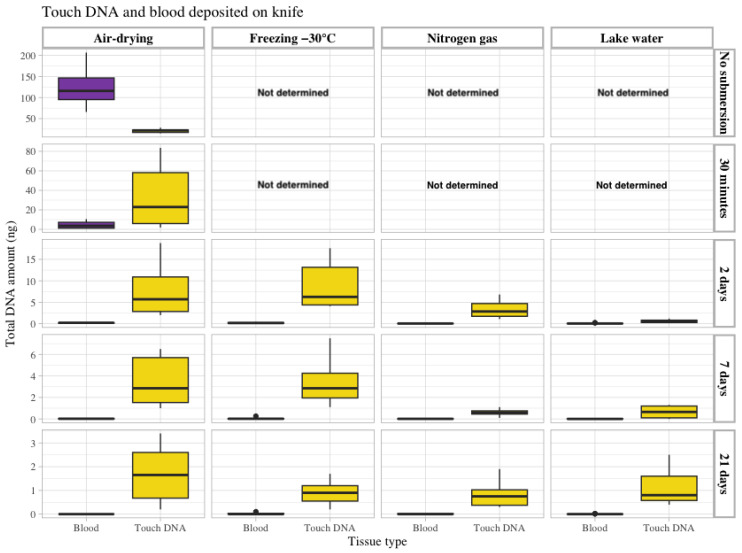
The total amount of recovered DNA in ng (Y-axis on the left) from touch DNA deposited on the knife shafts (yellow) and blood (50 µL) on the knife blades (purple). The data are grouped according to the four storage methods: air drying, freezing at −30 °C, nitrogen gas and lake water (upper X-axis) and the different submersion times included in the study: no submersion (dry reference), 30 min, 2 days, 7 days and 21 days (Y-axis on the right). Observe that the Y-axis (left) scale differs between submersion times.

Knives are often found and recovered by forensic divers and may be located in very different conditions. When the knives were recovered in the present study, some rust was observed on the blades after 2 days of submersion, which increased in quantity with longer submersion times. The total amount of DNA recovered from touch DNA and blood deposited on knives are shown in Figure 2. On average, 6-fold more DNA was retrieved from 50 µL of blood on the blade (average 122 ng) than from touch DNA deposited by holding the handle (average 21 ng) when the knives had not been submerged in water. After recovery from lake water, a general reduction in recovered DNA was observed with longer submersion time independent of the object storage method.

In contrast to the objects that were not submersed, the amount of DNA recovered from blood was less than from touch DNA, with significantly lower levels of DNA when comparing the same time intervals and storage conditions (shown in Table of *p*-values in Supplementary S1, Tables S2 and S5). This suggests blood is diffused in the lake water because a 27-fold decrease in DNA amounts was observed after just 30 min of submersion, and more extended exposure periods (2, 7 and 21 days) with objects submerged in water showed an even more negative effect on the DNA recovery for blood. In addition, for touch DNA on knives, the recovery of DNA was mainly affected by the time of submersion, with a 3-fold decrease observed after 2 days (air dried), and a 6-fold and 12-fold reduction in DNA contents was observed for 7 and 21 days in water, respectively.

In general, the highest amount of DNA was observed for both tissue types using the storage conditions of air-drying and freezing (Figure 2, Supplementary S1, Table S2). When evaluating the difference between storage conditions (with other parameters being constant), 19 of 21 conditions (seven samples/objects with submersion for 2, 7 and 21 days) yielded the highest average total recovered DNA for air-drying and freezing storage conditions. In contrast, 18 of 21 conditions yielded the lowest recovered DNA for nitrogen gas and lake water storage conditions (Figure 2 and Supplementary S1, Table S2).

**Figure 3:**
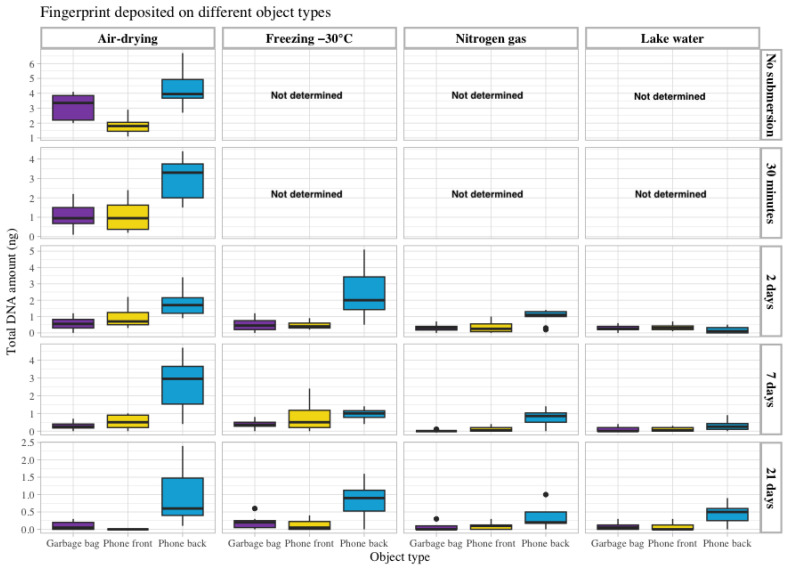
The total amount of DNA recovered in ng (Y-axis on the left) from a fingerprint deposited on three different objects: touch DNA deposited on a piece of a garbage bag (purple) and on the front (yellow) and back side (blue) of a smartphone. The data are grouped according to the four storage methods: air drying, freezing at −30 °C, nitrogen gas and lake water (upper X-axis) and the different submersion times included in the study: no submersion (dry reference), 30 min, 2 days, 7 days and 21 days (Y-axis on the right).

Touch DNA from a fingerprint of the same donor was deposited on four different types of objects with varying surface materials: a garbage bag made of soft plastic, the front of a smartphone made of glass, the back of the smartphone made of hard plastic and under a piece of duct tape. Thus, the recovery from different surfaces could be investigated and compared. Again, a general decrease in recovered DNA was observed with longer submersion time. However, as shown in Supplementary S1, Table S5, the difference in recovered DNA from the index finger deposited on the front side of the mobile phone made of glass and the soft plastic surface of the garbage bag was similar for most of the storage conditions. Approximately 2–4 times more DNA was recovered from the hard plastic surface on the back of the smartphone than from the glass surface on the front side. The lowest amount of DNA recovered was obtained from the garbage bag. Overall, the air-drying and freezing storage methods facilitated DNA recovery from these surface types (Figure 3). The results obtained for the fingerprint placed on a garbage bag and covered with packing tape showed a significantly higher amount of DNA recovered than for the other fingerprints (Supplementary S2).

The amount of DNA recovered from the hair samples was generally lower than other tissue types and showed considerable variation between biological replicates. Hair has different growth phases, and roots may be lacking for some of the biological replicates, possibly resulting in some high quantification results, which may be considered outliers. Although the amount of DNA recovered varied, a general decrease with longer submersion time was observed. The air-drying and freezing storage methods appeared to have the least negative impact on DNA recovery. Although a correlation between longer submersion times and low DNA amounts in combination with high degradation was observed for all object and tissue types, it still needs to be determined whether a downstream analysis of STR markers or mtDNA will be successful.

### 3.2. STR Genotyping

To evaluate the effect of lake water on DNA profiling, STR analysis was performed on one of the biological replicates for each of the evaluated conditions (seven sample/object types, three submersion times and four storage conditions). A subset of 98 samples was analysed, and the sample selection was based on the median DNA concentration value for each condition. The replicate closest to the median value was chosen for analysis. The selected replicates are shown in Supplementary S2 for all sample/object types and whether full or partial profiles were obtained. In addition, Supplementary S1, Table S3 provides an overview of the obtained STR profiles for the 21 analysed autosomal STR markers and displays the interpreted allele peaks and their corresponding peak heights. Due to the nature of these samples, only three of the seven sample/object types that were not submerged in water or submersed for 30 min resulted in complete STR profiles. Overall, the 84 samples submerged for 2, 7 or 21 days revealed that 11% of the samples had ≥75% of the alleles amplified, and 8% had ≥50% of the alleles amplified. The remaining 81% of the samples had ≤50% of amplified alleles. The most successful samples were touch DNA (holding the handle) on the knife and the fingerprints under the packing tape (Supplementary S1, Table S3).

In agreement with DNA quantification data, the shorter the water exposure time, the higher the possibility of obtaining interpretable STR profiles. Thus, the success rate in obtaining an STR profile was higher for the higher DNA concentration in the samples. For the blood deposited on a knife, complete STR profiles were generated for the no-submersion and 30 min submersion groups. Conversely, more extended submersion times (2 and 7 days) resulted in ≤50% of the expected alleles successfully amplified, with practically no alleles amplified after 21 days of submersion. The same trend was observed for touch DNA on the knife. However, 17–95% of the alleles were amplified after 2 days, 14–60% amplified after 7 days of submersion and 0–7% amplified after 21 days of lake water submersion.

**Figure 4:**
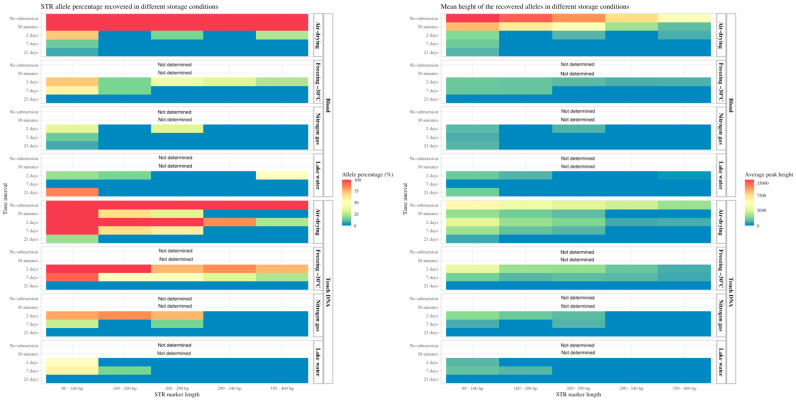
On the left: the percentage of STR alleles obtained from blood and touch DNA deposited on a knife and the recovered objects stored in different conditions (Y-axis right). The data are grouped according to the submersion time (Y-axis left). The percentage of positive STR alleles (concordant with the sample donor) is divided by a gradient scale (red bar for 100% of the alleles and blue bar for 0% of the alleles obtained. The STR marker length (X-axis) is also displayed. On the right: the mean peak heights (X-axis) of the obtained alleles in a gradient scale (red bar for an average peak height of 15,000 relative fluorescent units (RFU) and a blue bar for 0 RFU).

Regarding the four different storage conditions, air-drying resulted in 95% and 60% of the alleles amplified after 2 and 7 days of submersion, respectively. Similar success rates were observed for the freezing storage condition, with 88% and 60% of the alleles amplified after 2 and 7 days, respectively (Supplementary S1, Table S3). The overall success rates of the STR analysis for blood and touch DNA on the knife are illustrated in heatmaps in Figure 4, in which the shorter STR markers resulted in higher amplification success rates. The air-drying and freezing storage of objects after 2 and 7 days of water exposure had less DNA degradation because longer alleles were successfully amplified and higher peak heights were obtained.

**Figure 5:**
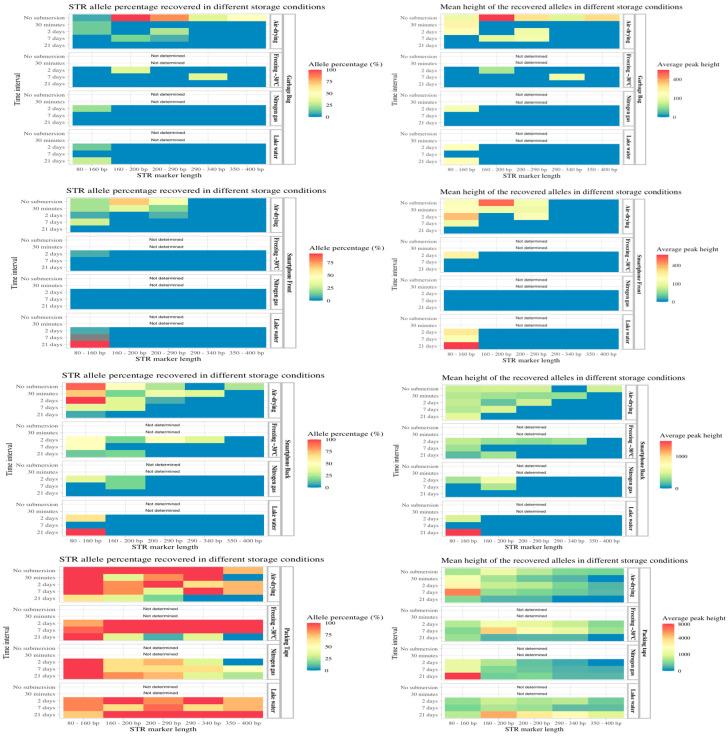
On the left: the percentage of STR alleles obtained from fingerprints deposited on different objects: garbage bag, front side of smartphone, back side of smartphone and adhesive side of packing tape, stored in different conditions (Y-axis right). The data are grouped according to the submersion time (Y-axis left). The percentage of positive STR alleles (concordant with the sample donor) is divided by a gradient scale (red bar for 100% of the alleles and blue bar for 0% of the alleles obtained. The STR marker length (X-axis) is also displayed. On the right: the mean peak heights (X-axis) of the obtained alleles in a gradient scale (red bar for an average peak height of 5000 relative fluorescent units (RFU) and a blue bar for 0 RFU).

For the fingerprints deposited on garbage bags and the glass on the front side of smartphones, <12% of the STR alleles were obtained after 2, 7 and 21 days of submersion (Supplementary S1, Table S3). The amplified alleles were mainly the miniSTRs with shorter amplicon lengths, with slightly more alleles of longer lengths obtained for the air-drying and freezing storage conditions (Figure 5). For fingerprints on the hard plastic surface on the back side of the smartphone, the freezing storage method was the most effective, resulting in the amplification of 55% of the alleles compared with 31% for the airdrying storage method after 2 days of submersion. After 7 days of submersion, the air-drying storage method generated 26% STR alleles, and the freezing storage method resulted in 11% of the STR alleles being amplified. In general, lake water and nitrogen gas as storage methods resulted in fewer recovered STR alleles from fingerprints on phones and garbage bags. In contrast, for the fingerprint under packing tape, a high number of alleles was amplified for all exposure times and storage methods. For this sample group, complete or almost complete STR profiles were obtained after 2, 7 and 21 days of water exposure. In general, the number and peak heights of the obtained alleles were high (>1000 RFUs for the complete STR profiles) for this group, indicating that tape over a fingerprint protects the DNA from diffusion into the water and degradation. After 2 and 7 days of submersion, the freezing method for storage yielded complete profiles with long and short fragments. Thus, both the time submerged in water and the storage conditions after retrieval of the objects have a significant effect on DNA quantity and the following DNA profiling. The statistical analysis showed the DNA concentration was significantly affected by submersion time in water and the different storage conditions (Supplementary S1, Table S5). In general, the duration of submersion in water affected the amount of DNA and STR analysis success, highlighting the importance of retrieving forensic evidence in water as soon as possible.

### 3.3. mtDNA Sequencing

The hair samples did not yield interpretable STR profiles for any of the storage methods or submersion times (Supplementary S1, Table S3). Therefore, analysis of mtDNA was performed on all hair samples (n = 56) using Sanger sequencing. Additionally, the biological replicates with the lowest concentration after 2, 7 and 21 days of submersion (n = 72) for types of objects with biological materials were Sanger-sequenced. Furthermore, the samples in which <75% of the STR alleles were amplified (n = 70) from the initial 84 samples analysed (excluding hair) were mtDNA analysed. The mtDNA sequencing of all hair samples (100%) was successful, as shown in Supplementary S1, Table S4. Among the 72 samples with the lowest DNA concentration, 45 samples (63%) yielded interpretable mtDNA sequence profiles. In addition, samples yielding < 75% of the STR alleles generated mtDNA profiles for 62 of 70 samples (89%). Notably, mtDNA sequences were obtained for all but one sample from hair, blood on the knife and fingerprints on the back side of the smartphone, independent of submersion time and storage methods. For all other biological sample/object types, the mtDNA results were negatively affected by longer exposure time. Regarding analysis of the samples with the lowest DNA concentration, the fingerprints on a garbage bag and the front side of the smartphone resulted in very few mtDNA sequences, and after 2 or 7 days of submersion, only samples from air-drying and freezing storage methods generated profiles. The overall success of the mtDNA analysis was 82% (163 of 198 samples) compared with 19% of STR profiles (16 of 84 samples), amplifying > 50% of the alleles.

## 4. Discussion

The present study was performed to evaluate the effects of lake water on DNA deposited on forensically interesting objects. Objects commonly found by forensic divers, such as knives, smartphones, tapes and garbage bags with DNA from fingerprints, blood and hair, were investigated. In total, 280 items (56 of each object type) with 392 DNA samples were submerged in water for different time periods. The samples were kept under four storage conditions before sampling (DNA swabbing or hair collection). The water exposure periods were 2, 7 and 21 days, and the evaluated storage conditions included air-drying, freezing, nitrogen gas and lake water from the dive site. The Quantifiler Trio Quantification Kit was used to estimate the amount of recovered DNA, which generally decreased with longer submersion time. The analysis was also used to evaluate the degradation (DI), a value calculated using the efficiency difference in amplifying two autosomal targets of different sizes. In general, the obtained DNA quantities decreased, and the DI values increased with longer submersion time.

STR analysis and sequencing of mtDNA were performed to evaluate the quantity and quality of the DNA. Both the STR and mtDNA analysis showed results that were concordant with the sample donors when the quantity and quality of the DNA from submersed objects revealed full profiles. The STR analysis data showed samples with longer submersion times tended to result in fewer successful profiles. An increased degradation with more extended submersion periods was demonstrated because only shorter STR loci (80–160 bp long), also known as miniSTRs, were obtained. Moreover, the longer the target, the more allelic drop-outs were observed, and the peak heights were lower for the amplified loci. The quality and quantity of recovered DNA rapidly decreased over time. The mtDNA analysis was more successful than the STR analysis due to the large copy number of the mtDNA genome in each cell, offering an alternative approach when STR profiling fails. Overall, the DNA quantification data, the success rate of STR and mtDNA analyses showed a negative correlation with exposure time. Therefore, forensic divers should recover evidence as soon as possible to ensure optimal DNA analysis results.

The DNA recovery was further evaluated based on the four storage conditions before sampling, and the air-drying and freezing methods yielded the highest DNA recovery (Supplementary S1, Table S2). Concordant with the higher DNA quantities, the STR and mtDNA analyses generated more DNA profiles from the air-drying and freezing storage methods than the other two storage conditions. These two storage methods are practical, easy to use and can be easily implemented by forensic divers. Optimal handling of objects between the collection from water and subsequent sampling is crucial. However, this process may be restricted by several factors, such as the size of equipment, the need for electricity, time consumption and cost. The storage condition most commonly used in Sweden is to place the object in water from the dive site until sampling and forensic analysis, mainly to limit the risk of damage to IT equipment and fingerprint traces. However, because less DNA was obtained from this storage condition than other storage conditions, implementing an updated storage method would improve DNA results. Significant variations were observed between tissue types and surface materials. For example, the different DNA sample types deposited on the knives showed a considerable variation in DNA recovery. Although about 6-fold more DNA was deposited with blood, significantly less DNA was recovered than from touch DNA. An explanation is that blood dissolves and spreads easily in water, as indicated by the reduced DNA amounts obtained after a short submersion time of 30 min (Supplementary S1, Table S2). This will also pose a risk of cross-contamination between samples in the water during the recovery of objects and transportation when objects have biological materials from several individuals or several objects are found together. In contrast, the skin cells may attach better to the surface due to a simultaneous deposition of fatty acids and other sebaceous components when touching the object.

Furthermore, a comparison of the amount of DNA recovered between fingerprints deposited on the different sides of a smartphone revealed the back of the phone (hard plastic) resulted in higher DNA recovery, indicating the DNA deposited on the plastic was less affected by submersion in water than DNA deposited on the front side (glass). This suggests the surface features (e.g., porous, non-porous, compact, textured) of different surfaces attach and retain cells differently [2,17]. A similar observation was reported in a study by Castelló et al. [18], in which fewer STR alleles were obtained from DNA on a glass surface than from a metal surface on items recovered from stagnant tap water. The choice of DNA extraction method will also affect the results, and in this study, the Chelex^®^ 100 Resin was used for extracting DNA. Although this is a quick method with few steps, other methods, for example, the QIAamp^®^ DNA Investigator Kit or DNeasy^®^ Blood & Tissue Kit, are likely better to recover DNA in high quantity and free of inhibitors.

In the present study, the DNA samples from fingerprints on the adhesive side of the tape were less affected by time or storage conditions. The tape retained more touch DNA than other objects, possibly due to being protected between the tape and the garbage bag and better attached to the glue. Thus, the adhesive part of the tape covers the cellular material and DNA in a water environment. This agrees with a study by Forger et al. [9], in which the authors suggested the adhesive material of the tape protects the DNA subjected to aqueous conditions. The hair samples resulted in very poor STR results from the submersed samples, but complete mtDNA profiles could be obtained for all hairs. Although hair has a low cell count and the time in water appears to degrade the DNA, mtDNA profiles could be generated.

Variation was observed in DNA quantity, STR and mtDNA results even within groups prepared in the same manner and handled under the same conditions which may have been caused by several different factors. Determining the exact amount of touch DNA to be deposited on each object is difficult. The deposition process can be designed to leave similar amounts of DNA (equal pressure, area, finger used). However, variation in deposited and recovered DNA can be expected. This might explain why more touch DNA, 32.2 ng on average, was recovered after 30 min of submersion compared with 21.3 ng for samples that were not submersed (Supplementary S1, Table S2). Furthermore, DNA sampling may be more challenging from some materials, such as tape attached to plastic bags, when the sticky glue is difficult to dissolve. Thus, a considerable variation between the samples from the same object and storage condition was observed for touch DNA. Nevertheless, all evaluated materials in the present study are relevant because forensic divers often find them, and knowing possible variations exist is essential and should be considered in forensic casework analysis.

In summary, the DNA quantification results showed that the recovered DNA was negatively affected and proportionally decreased with the water exposure time. Furthermore, a general increase in degradation of the DNA was observed with longer water exposure time. To our knowledge, this is the first study in which different storage methods for samples recovered from water were evaluated. Although the research indicated that freezing and air-drying storage methods could result in more recovered DNA than nitrogen gas and lake water storage methods, further studies are needed. For example, the effects on forensic analyses other than DNA profiling, such as fingerprint identification, ballistics and IT forensics, must also be evaluated. In a previous study, latent fingerprints could be visualised when using enhancement chemicals after 1, 2 or 10 days of submersion in water. However, the fingerprint detection decreased with submersion time [19]. The possibility of identifying individuals based on DNA, latent fingerprints or other forensic analyses will often depend on the duration of water exposure, the dynamic aquatic environment and the object’s surface. In addition, the different types of water (sea, river, lake or ocean), climate and other environmental factors in the area are likely to be essential and warrant further investigation. Increased knowledge regarding forensic analyses of items found in water will aid in developing optimal conditions for retrieval, storage and analysis of these challenging samples and ultimately generate more forensic evidence to identify more suspects, ultimately leading to more convictions in court.

## Data Availability

Data are contained within the article and Supplementary Materials.

## Supplementary Materials

The following are available online at https://www.mdpi.com/article/10.3390/genes15030279/s1, Supplementary S1, Table S1. Number and overview of objects submerged in water for the different time intervals studied; Table S2. Average of total DNA quantity in ng obtained from all biological materials and standard deviations excluding and including outliers in all the different conditions; Table S3. Amplification results of 21 autosomal STR markers using the GlobalFiler Kit organised based on the marker size and peak height data; Table S4. Overview of the mtDNA amplification of HV1 (16128–16348) of selected samples from the biological materials closest to the median concentration and with the lowest concentration, as well as all hair samples; Table S5. Table of *p*-values resulting from the investigation of significant differences between the quantification values in all possible conditions using the Wilcoxon test in R; Supplementary S2, Tables S6–S12. Overview of the amount of DNA recovered for all samples based on the object and biological material deposited.
